# Maternal Prkce expression in mature oocytes is critical for the first cleavage facilitating maternal‐to‐zygotic transition in mouse early embryos

**DOI:** 10.1111/cpr.13231

**Published:** 2022-05-18

**Authors:** Shaoqing Zhang, Xiuli Gong, Yiye Zhou, Qingwen Ma, Qin Cai, Guanheng Yang, Xinbing Guo, Yanwen Chen, Miao Xu, Yiwen Zhu, Yitao Zeng, Fanyi Zeng

**Affiliations:** ^1^ School of Life Sciences and Biotechnology & Shanghai Children's Hospital Shanghai Jiao Tong University Shanghai China; ^2^ Department of Histo‐Embryology, Genetics and Developmental Biology Shanghai Jiao Tong University School of Medicine Shanghai China; ^3^ NHC Key Laboratory of Medical Embryogenesis and Developmental Molecular Biology & Shanghai Key Laboratory of Embryo and Reproduction Engineering Shanghai China; ^4^ School of Pharmacy Macau University of Science and Technonlogy, Taipa Macau China

## Abstract

**Objectives:**

Early embryo development is dependent on the regulation of maternal messages stored in the oocytes during the maternal‐to‐zygote transition. Previous studies reported variability of oocyte competence among different inbred mouse strains. The present study aimed to identify the maternal transcripts responsible for early embryonic development by comparing transcriptomes from oocytes of high‐ or low‐ competence mouse strains.

**Materials and Methods:**

*In vitro* fertilization embryos from oocytes of different mouse strains were subject to analysis using microarrays, RNA sequencing, real‐time quantitative PCR (RT‐qPCR) analysis, Western blotting, and immunofluorescence. One candidate gene, *Prkce*, was analysed using *Prkce* knockout mice, followed by a cRNA rescue experiment.

**Results:**

The fertilization and 2‐cell rate were significantly higher for FVB/NJ (85.1% and 82.0%) and DBA/2J (79.6% and 76.7%) inbred mouse strains than those for the MRL/lpr (39.9% and 35.8%) and 129S3 (35.9% and 36.6%) strains. Thirty‐nine differentially expressed genes (DEGs) were noted, of which nine were further verified by RT‐qPCR. *Prkce* knockout mice showed a reduced 2‐cell rate (*Prkce* ^*+/+*^ 80.1% vs. *Prkce* ^*−/−*^ 32.4%) that could be rescued by *Prkce* cRNA injection (2‐cell rate reached 76.7%). Global transcriptional analysis revealed 143 DEGs in the knockout mice, which were largely composed of genes functioning in cell cycle regulation.

**Conclusions:**

The transcription level of maternal messages such as *Prkce* in mature oocytes is associated with different 2‐cell rates in select inbred mouse strains. *Prkce* transcript levels could serve as a potential biomarker to characterize high‐quality mature oocytes.

## Graphical abstract

Oocyte competence is variable among different mouse strains, most likely due to the differential abundance of maternal transcripts in the oocyte during MZT. One candidate gene, Prkce, plays an essential role in facilitating MZT, especially during the first cleavage. The expression level of the *Prkce* transcript appears to be associated with different oocyte competence in different mice strains.

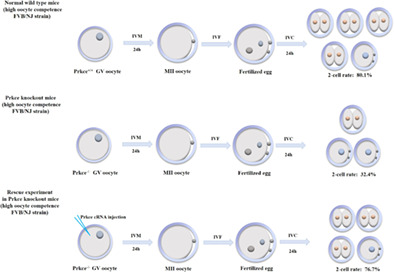

## 1 INTRODUCTION

Maternal‐to‐zygotic transition (MZT) is a process in which newly generated zygotic mRNAs replace maternal mRNAs accumulated in oocytes during zygotic genome activation (ZGA). After MZT, development control is switched from maternal genes to zygotic genes.^1^ ZGA mainly occurs at a late stage of 2‐cell embryos in mice. There are only minute amounts of de novo transcription during the 1‐cell stage.^2^, ^3^, ^4^ Mature oocytes are known to store almost all maternal mRNA required for fertilization, the first cleavage, and ZGA.

At present, several maternal genes have been revealed to participate in early embryonic developmental regulation, including the well‐known *Mater* and *Npm2*, which play important roles in ZGA and chromosomal remodelling, respectively.^5^, ^6^ However, many maternal transcripts involved in regulating early embryonic development remain unknown. The application of high‐throughput methods allows us to compare the transcriptome between the oocytes with high or low developmental potential to reveal more maternal transcripts responsible for early embryonic development. Recently, using high‐throughput methods, several studies have investigated maternal transcripts affecting embryonic developmental outcome involving follicle size, female age, oocyte maturation modes (*in vitro* and *in vivo*), chromatin configuration, and so on, in a variety of species.^7^

One previous study showed significant variability in developmental outcome at the 2‐cell stage among mature oocytes derived from different inbred mouse strains.^8^ It is important to note that there are many single nucleotide polymorphisms among mouse strains,^9^ which have the potential to lead to differing patterns of maternal gene expression. It is important to explore what patterns of maternal transcripts in mature oocytes facilitate embryonic development through the 2‐cell stage.

In the present study, mature oocytes derived from four highly inbred mouse strains were inseminated with the same batch of ICR mouse sperm using *in vitro* fertilization (IVF). The fertilization rate and 2‐cell rate of FVB/NJ and DBA/2J oocytes and those of MRL/lpr and 129S3 oocytes were documented, and differentially expressed genes (DEGs) from global expression profiles were analyzed. All identified candidate genes were verified by real‐time quantitative PCR (RT‐qPCR). Subsequently, one promising candidate gene, *Prkce*, was further analyzed, and functional studies were performed using *Prkce* knockout mice. *Prkce* was shown to act to help to facilitate the MZT in mouse early embryos at the first cleavage.

## 2 MATERIALS AND METHODS

### 2.1 Mice feeding and ethics statement

All mice, including four inbred strains of 6–8 weeks old female mice (FVB/NJ, DBA/2J, MRL/lpr, and 129S3) and 3–6 months old ICR male mice, were purchased from Shanghai SLAC Laboratory Animal Co. (Shanghai, China). All mice were housed under temperature‐controlled (22°C) conditions and the light cycle was set at 12 h on and 12 h off. Water and feed were provided ad libitum. All experimental animal protocols were approved by the Institutional Animal Care and Use Committee of the Shanghai Children's Hospital and carried out following the regulations drafted by the Institutional Animal Care and Use Committee of the Shanghai Children's Hospital.

### 2.2 Oocyte collection

Oocytes were collected from 6 to 8 weeks old female mice. Female mice were given an intraperitoneal (IP) injection of 5 IU pregnant mare serum gonadotrophing (PMSG) (Ningbo Second Hormone Factory). To collect germinal vesicle (GV) stage oocytes, mice were sacrificed by cervical dislocation 48 h after PMSG injection to harvest the ovaries. GV oocytes were collected from the ovaries by puncturing follicles with a needle of a 1 ml injector in M2 medium with 2.5 μM milrinone (Abcam). To collect MII stage oocytes, an IP injection with 5 IU of human chorionic gonadotropin (hCG) (Livzon) 48 h after PMSG injection was performed. MII oocytes were collected 14 h after hCG injection from the ampulla of the oviducts in M2 medium. In RNA extraction experiments, cumulus cells were removed by treatment with 300 IU/ml hyaluronidase (Sigma‐Aldrich) in M2 medium for 2 min, and the oocytes were picked out by mouth pipette and rinsed in M2 medium at least five times under stereomicroscope (Olympus SZH‐ILLK). The MII oocytes were picked out and stored at −80°C immediately until use.

### 2.3 *In vitro* maturation

GV stage oocytes were cultured in *in vitro* maturation (IVM) medium consisted of JY IVM Medium (ARSCI) supplemented with 75 mIU/ml of follicle‐stimulating hormone (FSH) (Ningbo Second Hormone Factory), 75 mIU/ml of luteinizing hormone (LH) (Ningbo Second Hormone Factory) for 24 h in the incubator (5% CO_2_ in air, 37°C) to obtain MII oocytes. The rate of mature oocytes was recorded.

### 2.4 *In vitro* fertilization

For IVF, ICR male mice of 3–6 months old were sacrificed by cervical dislocation. Caudae epididymides were collected and placed into 400 μl Sydney IVF Fertilization Medium (Cook), which was covered with mineral oil (Sigma‐Aldrich) and pre‐balanced for at least 4 h in the incubator (5% CO_2_ in air, 37°C). The caudae epididymides were cut five times, squeezed gently using scissors, and returned to the incubator for 10 min to allow the spermatozoa to swim out. Following this procedure, the caudae epididymides were removed from the solution. The spermatozoa concentration was determined using a sperm counting plate. The spermatozoa were transferred to 100 μl Sydney IVF Fertilization Medium to make a fertilization drop with final concentration of 1 × 10^6^ sperm per ml. Spermatozoa were capacitated for a total of 1.5 h before IVF, at which time the oocytes were transferred into the fertilization drop for insemination in the incubator. Four hours later, the inseminated oocytes were washed with M2 medium to remove redundant spermatozoa. Only the MII oocytes with normal appearance were transferred to a minimal volume of KSOM medium drop covered with mineral oil that was previously equilibrated for at least 4 h in the incubator for further culture. The number of fertilized eggs and 2‐cell embryos was recorded after 24 h culture.

### 2.5 RNA extraction, amplification, and microarray analysis

Total RNA was extracted from each sample containing 30–50 MII oocytes using PicoPure RNA Isolation Kit (ThermoFisher Scientific) following the manufacturer's instructions and treated with DNase I (QIAGEN) to eliminate DNA contamination. The quality of total RNA was determined using Agilent RNA 6000 Pico Kit (Agilent Technologies) on Agilent 2100 bioanalyzer (Agilent Technologies) according to manufacturer's protocol. The RNA was used in the microarray and RT‐qPCR assays with the criteria of RNA integrity number (RIN) ≥7.0 and 28S/18S >0.7.

According to the user manual, 3 ng total RNA of the oocytes from each strain were processed using GeneChip 3′ IVT Pico Kit (ThermoFisher Scientific) and then were applied to GeneChip Mouse Genome 430 2.0 Array (ThermoFisher Scientific). Arrays were scanned using GeneChip Scanner 3000 to generate. CEL intensity files.

Microarray analysis was performed as described in detail previously.^2^ Briefly, raw data (.CEL files) were processed using the MAS 5.0 (Affymetrix Expression Console software) followed by one‐way ANOVA (unpaired) analysis (Affymetrix Transcriptome Analysis Console 2.0 software). *p*‐values were adjusted using Bonferroni's method to control the error rate. A gene was declared to be significantly differentially expressed if its adjusted *p*‐value was <0.05 (Bonferroni correction), absolute fold change was >2, and a false discovery rate (FDR) <0.05. DEGs were ranked using hierarchical clustering using Affymetrix Transcriptome Analysis Console 2.0 software.

### 2.6 Gene Ontology and quantitative trait locus analysis of DEGs

Differentially expressed genes were performed Gene Ontology (GO) analysis with DAVID Bioinformatics Resources 6.8 (https://david.ncifcrf.gov/) as previously described.^2^ Enriched biological processes and molecular functions with *p* < 0.05 were considered as statistically significant. In parallel, the cellular component analysis was carried out by DAVID Bioinformatics Resources 6.8 to investigate the location of the proteins encoded by DEGs. Summary of DEGs was obtained from the NCBI GENE database (http://www.ncbi.nlm.nih.gov/gene/). Furthermore, their position analysis relative to reproductive system quantitative trait locus (QTLs) was performed on MGI (http://www.informatics.jax.org/).

### 2.7 Real‐time quantitative PCR analysis

Total RNA was primed with 1 μl oligo dT (12–18) primer (50 μM) in a 6‐μl reaction system, and then incubated at 70°C for 10 min and rapidly cooled on ice prior to reverse transcription reaction. Reverse transcription was carried out at 42°C for 1 h in a final volume of 10 μl containing 2 μl of 5× M‐MLV buffer (TAKARA BIO), 0.5 μl of 10 mM dNTP mix (NEW ENGLAND Biolabs), 0.25 μl of RNase Inhibitor (TAKARA BIO), 1 μl of RTase M‐MLV (RNase H‐) (TAKARA BIO) followed by incubation at 70°C for 15 min to terminate the reaction and stored at ‐20°C until use. For real‐time PCR, the cDNA samples were diluted tenfold.

RT‐qPCR was conducted on LightCycler 96 (Roche). Each reaction mixture consisted of 2 μl of cDNA, 0.5 μl each of forward (10 μM) and reverse primers (10 μM), 12.5 μl 2× SYBR Premix Ex TaqTM II (Tli RNaseH Plus) (TAKARA BIO), and 9.5 μl of nuclease‐free water (Millipore) in a total reaction volume of 25 μl. The thermal cycler program consisted of 40 cycles of 95°C for 15 s and 60°C for 1 min. Primers were designed using Primer Premier 5.0 (PRIMIER Biosoft). The primer sequences are listed in Table S1. Data were obtained from three replicate assays for each sample with normalization to ActB, and relative gene abundance was calculated using the method of 2^−ΔΔCt^ as previously described.^10^

### 2.8 Generation of *Prkce* knockout mice

In order to generate the knockout *Prkce* mice using CRISPR‐Cas9 technology in this study, we designed the guide RNA 1 (target site: aacgtggacgactcgcgcat) and 2 (target site: tatcggctacgacgacttcg) to specifically target the exon 1 of *Prkce* with an online design tool (http://crispr.mit.edu/). The dsDNA encoding guide RNA was cloned using Invitrogen™ GeneArt™ CRISPR Nuclease Vector with OFP Reporter Kit (ThermoFisher Scientific). Guide RNA was transcribed *in vitro* using Invitrogen MEGAshortscript™ T7 Transcription Kit (ThermoFisher Scientific), and Cas9 mRNA was transcribed using Invitrogen GeneArt CRISPR Nuclease mRNA kit (ThermoFisher Scientific). The guide RNA and Cas9 mRNA were purified with Invitrogen MEGAclear™ Transcription Clean‐Up Kit (ThermoFisher Scientific). All the above experimental procedures were performed according to the user manual. The guide RNA 1 (50 ng/μl) and 2 (50 ng/μl) were co‐injected with Cas9 mRNA (100 ng/μl) into the cytoplasm of fertilized eggs from FVB/NJ mice and the injected eggs were transferred to pseudopregnant ICR females.

The pups were genotyped by PAGE‐PCR assays. In brief, the regions spanning the guide RNA 1 and 2 target sites were amplified by PCR using primers F: 5′‐AACGGACGTCTCCAGCTCTC‐3′ and R: 5′‐GCAAGTCTTTCCCTGGGACC‐3′. The mutant pups were determined by DNA sequencing, and the identified *Prkce*‐deficient mice were used as a founder to generate the transgenic breeding colony.

### 2.9 Immunoblot analysis

For Western blotting, the ovaries were collected in RIPA Lysis Buffer (Beyotime) supplemented with 1 mM phenylmethanesulfonyl fluoride (Beyotime) and protease inhibitor cocktail (Sigma‐Aldrich). Lysates were separated with sodium dodecyl sulfate‐polyacrylamide gel electrophoresis (BIO‐RAD) and electrophoretically transferred onto polyvinylidene fluoride membrane (BIO‐RAD). After blocking with Tris‐buffered saline supplemented with Tween 20 (TBST) containing 5% skim milk (BD Biosciences) for 1 h, the membrane was incubated overnight at 4°C with the primary antibody targeting PKCE (1:500) (ThermoFisher Scientific) and ACT β (1:25,000) (Proteintech). After being washed three times in TBST for 10 min, membranes were incubated for 1 h at 37°C with secondary antibodies, polyclonal Goat Anti‐Rabbit Immunoglobulins/horseradish peroxidase (HRP; 1:5000; Agilent) or Polyclonal Rabbit Anti‐Mouse Immunoglobulins/HRP (1:5000; Agilent). After washing three times in TBST for 10 min, blots were developed using Clarity™ Western ECL substrate (BIO‐RAD). Protein bands were visualized using Amersham Imager 600 (GE Healthcare).

### 2.10 Immunofluorescence

For immunofluorescence, MII oocytes were fixed with 3.7% paraformaldehyde (Sigma‐Aldrich) in phosphate‐buffered saline (PBS) for 20 min and permeabilized with permeabilization solution (0.1% v/v Triton X‐100, 0.3% w/v BSA, 0.01% Tween 20 in PBS) for 20 min at room temperature. The oocytes were placed in blocking solution (0.3% w/v BSA, 0.01% Tween 20 in PBS) for 1 h at room temperature, and then incubated with Anti‐PKC epsilon antibody (1:200; Abcam) for 1 h followed with Goat Anti‐Rabbit IgG H&L (Alexa Fluor® 488) preadsorbed (1:500; Abcam) for 1 h at room temperature. Nuclei were counterstained with fluoroshield mounting medium with 4′,6‐diamidino‐2‐phenylindole (Abcam). Images were acquired by Leica TCS SP5 II Confocal Microscope (Leica).

### 2.11 Production of *Prkce* cRNA and microinjection

Microinjections were performed using a microinjector (Eppendorf). To obtain *Prkce* cRNA, Prkce (NM_011104) Mouse Tagged ORF Clone (OriGene) was linearized using Ase I (NEW ENGLAND Biolabs). HiScribe T7 ARCA mRNA kit (NEW ENGLAND Biolabs) was used to produce 5′ capped and 3′ polyA‐tailed mRNA, purified using an Invitrogen MEGAclear Transcription Clean‐Up Kit (ThermoFisher Scientific). The concentration of *Prkce* cRNA was determined using Nanodrop 2000 (ThermoFisher Scientific). The *Prkce* cRNA solution (671 ng/μl) or nuclease‐free water was microinjected into the cytoplasm of GV oocytes in an M2 medium drop supplemented with 2.5 μM milrinone. After microinjection, the oocytes were washed in M2 and matured *in vitro*.

### 2.12 RNA sequencing and DEGs identification

Twelve mature oocytes in each sample were obtained by IVM and collected for RNA sequencing. Library construction and whole‐transcriptome sequencing were performed by Genergy Biotechnology, Inc. In brief, mRNA amplificated by the Single Cell Full Length mRNA Amplication Kit (Vazyme Biotech) and sequencing libraries were constructed using TruePrep DNA library Prep Kit V2 for Illumina (Vazyme Biotech) according to the reagent preparation guide. The RNA sequencing was performed on the Illumina Nova6000 sequencer (Illumina). The expression levels for each gene were normalized to fragments per kilobase of transcript per million fragments mapped to compare mRNA abundance between samples. DEGs were identified using DESeq2 Bioconductor package, and significant DEGs were selected using a criterion of *p* < 0.05, FDR ≤0.05, and a log2 (fold change) ≥1. The DEGs were submitted for GO enrichment analysis with the Bioconductor software package topGO. Significant GO terms with *p* ≤ 0.05 were shown. The DEGs were also submitted for Kyoto Encyclopedia of Genes and Genomes (KEGG) analysis (https://www.kegg.jp/), and significant pathways with *p* ≤ 0.05 were shown.

### 2.13 Statistical analysis

The maturation, fertilization, and the first cleavage rates were compared between different groups using a chi‐square test. Using one‐way ANOVA, the relative abundance of mRNA was analyzed by SPSS 20.0 software (SPSS). A significant difference was defined when the *p*‐value was <0.05. All experiments were replicated at least three times, and the data were shown as mean ± SEM.

## 3 RESULTS

### 3.1 Comparison of fertilization rate and the first cleavage rate using mature oocytes derived from four strains of mice

In mice, embryonic development before ZGA is predominantly controlled by the initial pool of genes expressed in mature oocytes. Therefore, the strains with significant differences in fertilization rate and 2‐cell rate are a good model for investigating critical maternal transcripts.

In this study, mature oocytes from mice of FVB/NJ, DBA/2J, MRL/lpr, and 129S3 inbred strains were fertilized with the same batch of spermatozoa from ICR mice for IVF. The results showed that the fertilization rate and 2‐cell rate were significantly higher for FVB/NJ (85.1% and 82.0%) and DBA/2J (79.6% and 76.7%) oocytes than those for MRL/lpr (39.9% and 35.8%) and 129S3 (35.9% and 36.6%) oocytes (Table 1). Accordingly, FVB/NJ and DBA/2J were classified as high oocyte competence (HOC) group, while MRL/lpr and 129S3 were classified as low oocyte competence (LOC) group.

**TABLE 1:** *In vitro* fertilization results of mature oocytes derived from FVB/NJ, DBA/2J, MRL/lpr, and 129S3 mice

Mouse strain	No. mature oocytes	No.fertilized eggs (%)	No. 2‐cell embryos from fertilized eggs (%)
FVB/NJ	281	239 (85.1)^a^	196 (82.0)^a^
DBA/2J	285	227 (79.6)^a^	174 (76.7)^a^
MRL/lpr	363	145 (39.9)^b^	52 (35.8)^b^
129S3	510	183 (35.9)^b^	67 (36.6)^b^

*Note*: Values in the same column with different superscripts are significantly different.

### 3.2 Differences of oocytes transcriptome between HOC strains and LOC strains

**FIGURE 1:**
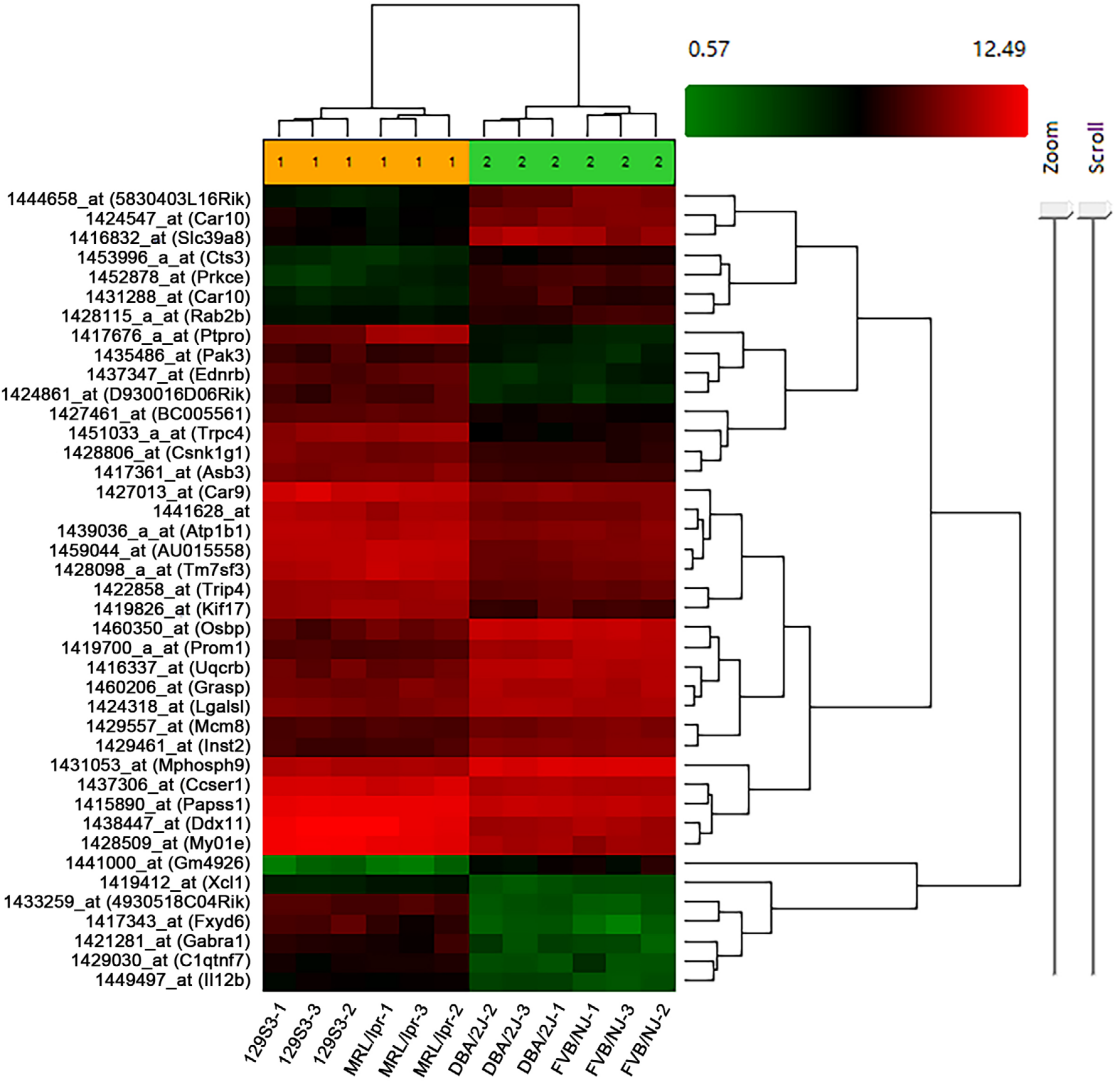
Clustering analysis based on the differentially expressed genes between low oocyte competence (LOC) group and high oocyte competence (HOC) group. 1 represents the LOC group; 2 represents the HOC group. Each sample contains 30–50 oocytes.

The transcriptional pattern of mature oocytes was compared between HOC and LOC strains. Three replicates of MII oocyte samples from each of the four inbred mouse strains were analyzed by microarrays with 45,037 probe sets representing over 34,000 well‐substantiated genes. By One‐Way Between‐Subject ANOVA (unpaired) method, 39 DEGs labelled by 41 probe sets were selected between HOC strains and LOC strains, among which 24 genes were upregulated, and 15 genes were downregulated in the LOC group, respectively (Figure 1). The complete DEGs are listed in Table 2.

**TABLE 2:** Differentially expressed genes in the mature oocytes between oocytes from the low oocyte competence group and high oocyte competence group

Gene symbol	Transcript cluster ID	Transcript ID	LOC vs. HOC		
			Fold change	*p*‐value	FDR *p*‐value
*LOC102638761*	1433259_at	Mm.158138.1	49.2	3.56E‐11	0.000002
*Fxyd6*	1417343_at	Mm.24808.1	49.06	1.06E‐07	0.00028
*C1qtnf7*	1429030_at	Mm.33391.1	18.19	2.51E‐07	0.000365
*Ptpro*	1417676_a_at	Mm.4715.1	18.15	8.07E‐07	0.000744
*Gabra1*	1421281_at	Mm.4914.1	15.66	3.14E‐07	0.000392
*Ednrb*	1437347_at	Mm.129235.1	12.43	6.87E‐08	0.000206
*Il12b*	1449497_at	Mm.997.1	10.84	3.19E‐08	0.00012
*D930016D06Rik*	1424861_at	Mm.31098.1	9.32	2.28E‐08	0.000114
*Trpc4*	1451033_a_at	Mm.10100.1	8.62	1.05E‐08	0.000079
*Pak3*	1435486_at	Mm.40035.1	5.52	9.40E‐07	0.000814
*Xcl1*	1419412_at	Mm.190.1	5.27	1.31E‐07	0.00028
*Kif17*	1419826_at	Mm.219916.1	4.46	1.18E‐07	0.00028
*Ddx11*	1438447_at	Mm.133459.1	4.1	1.41E‐07	0.000287
*Myo1e*	1428509_at	Mm.100720.1	3.52	3.35E‐07	0.000408
*BC005561*	1427461_at	Mm.35017.1	3.29	9.59E‐10	0.000022
*Tm7sf3*	1428098_a_at	Mm.18761.1	3.26	2.28E‐07	0.000342
*AU015558*	1459044_at	Mm.25921.1	3.09	1.71E‐07	0.000297
*Car9*	1427013_at	Mm.45576.1	3.04	2.64E‐07	0.000371
*Csnk1g1*	1428806_at	Mm.66616.1	2.97	7.51E‐08	0.000211
*Asb3*	1417361_at	Mm.24331.1	2.78	2.91E‐08	0.000119
*Trip4*	1422858_at	Mm.27462.1	2.46	2.60E‐08	0.000117
*Atp1b1*	1439036_a_at	Mm.4550.6	2.36	1.79E‐07	0.000298
*Papss1*	1415890_at	Mm.18161.1	2.02	3.88E‐09	0.000047
*Ccser1*	1437306_at	Mm.12522.1	2.01	7.12E‐07	0.000697
*Mcm8*	1429557_at	Mm.45710.1	−2.07	9.33E‐07	0.000814
*Lgalsl*	1424318_at	Mm.76694.1	−2.14	2.16E‐07	0.000337
*Mphosph9*	1431053_at	Mm.40818.1	−2.23	3.85E‐08	0.000124
*Grasp*	1460206_at	Mm.116916.1	−2.45	8.10E‐07	0.000744
*Ints2*	1429461_at	Mm.220929.1	−2.92	1.92E‐08	0.000114
*Uqcrb*	1416337_at	Mm.24805.1	−3.41	1.30E‐07	0.00028
*Rab2b*	1428115_a_at	Mm.32870.1	−3.58	4.78E‐07	0.000489
*Prom1*	1419700_a_at	Mm.6250.1	−4.71	9.63E‐09	0.000079
*Osbp*	1460350_at	Mm.87450.1	−4.77	3.12E‐07	0.000392
*Car10*	1431288_at	Mm.44999.1	−4.87	1.26E‐07	0.00028
*Cts3*	1453996_a_at	Mm.46079.2	−5.06	2.95E‐07	0.000391
*Car10*	1424547_at	Mm.94055.1	−7.31	2.17E‐07	0.000337
*Teddm2*	1444658_at	Mm.165790.1	−10.12	7.02E‐07	0.000697
*Prkce*	1452878_at	Mm.2013.1	−10.55	3.51E‐07	0.000416
*Slc39a8*	1416832_at	Mm.30239.1	−12.47	3.87E‐08	0.000124
*Gm4926*	1441000_at	Mm.33553.1	−30.94	1.67E‐07	0.000297

Abbreviations: FDR, false discovery rate; HOC, high oocyte competence; LOC, low oocyte competence.

Following this, GO enrichment analysis was performed and seven biological processes and four molecular functions showed a significant difference between the HOC group and LOC group, including ion transport, ATP binding, nucleotide binding, and ion channel activity (Table 3).

**TABLE 3:** Functional annotations of differentially expressed genes between oocytes from the low oocyte competence group and high oocyte competence group

Category	Gene ontology term	Genes	*p*‐value
Biological process	GO:0006810~transport	*Atp1b1*, *Fxyd6*, *Rab2b*, *Gabara1*, *kif17*, *Osbp*, *Slc39a8*, *Trpc4*, *Uqcrb*	0.005149
	GO:0072112~glomerular visceral epithelial cell differentiation	*Prom1*, *Ptpro*	0.010791
	GO:0006811~ion transport	*Atp1b1*, *Fxyd6*, *Gabara1*, *Slc39a8*, *Trpc4*	0.011913
	GO:0010763~positive regulation of fibroblast migration	*Pak3*, *Prkce*	0.027522
	GO:0001916~positive regulation of T cell‐mediated cytotoxicity	*Xcl1*, *Il12b*	0.029029
	GO:0032733~positive regulation of interleukin‐10 production	*Xcl1*, *Il12b*	0.035036
	GO:0030001~metal ion transport	*Atp1b1*, *Slc39a8*	0.038026
Molecular function	GO:0005524~ATP binding	*Papss1*, *Atp1b1*, *Ddx11*, *Csnk1g1*, *Kif17*, *Mcm8*, *Myo1e*, *Pak3*, *Prkce*	0.001517
	GO:0000166~nucleotide binding	*Papss1*, *Rab2b*, *Ddx11*, *Csnk1g1*, *Kif17*, *Mcm8*, *Myo1e*, *Pak3*, *Prkce*	0.007254
	GO:0005216~ion channel activity	*Fxyd6*, *Gabra1*, *Trpc4*	0.028248
	GO:0045296~cadherin binding	*Prom1*, *Trpc4*	0.043957

Differentially expressed genes relative to the QTLs for the reproductive system were further analyzed. Twelve DEGs are involved in the “reproductive” QTLs (Table 4), among which four genes (*Prkce*, *Cts3*, *Uqcrb*, *Ddx11*) were located in the “pregnancy” QTLs.

**TABLE 4:** The quantitative trait locus (QTL) location of differentially expressed genes between oocytes from the low oocyte competence group and high oocyte competence group

Gene symbol	QTL name (symbol)
*Asb3*	Ovulation rate QTL 2 (Orq2)
*Gm4926*	Ovulation rate QTL 2 (Orq2)
*Lgalsl*	Ovulation rate QTL 2 (Orq2)
*Gabra1*	Ovulation rate QTL 2 (Orq2)
*Il12b*	Ovulation rate QTL 2 (Orq2)
*Cts3*	Pregnancy QTL 3 (Pregq3)
*Uqcrb*	Pregnancy QTL 3 (Pregq3)
*Ddx11*	Pregnancy QTL 4 (Pregq4)
*Prkce*	Pregnancy QTL 4 (Pregq4)
*Pak3*	Sperm head anomaly 3 (spha3) testis weight (Tswt)
*Tm7sf3*	Testis weight QTL 2 (Tesq2)
*Ptpro*	Testis weight QTL 2 (Tesq2)

### 3.3 RT‐qPCR verification of candidate DEGs

Another four batches of mature oocytes from the four mouse strains independent of the microarray samples were collected, and the transcription profiles of 11 DEGs were verified by RT‐qPCR. Among these genes, four (*Atp1b1*, *Uqcrb*, *Fxyd6*, and *Trpc4*) are associated with transportation, five genes (*Prkce*, *Ddx11*, *Papss1*, *Myo1e*, and *Mcm8*) are associated with ATP binding, and two genes (*Car9* and *Car10*) belong to the same carbonic anhydrases family. Their functions with respect to reproduction remain unclear.

**FIGURE 2:**
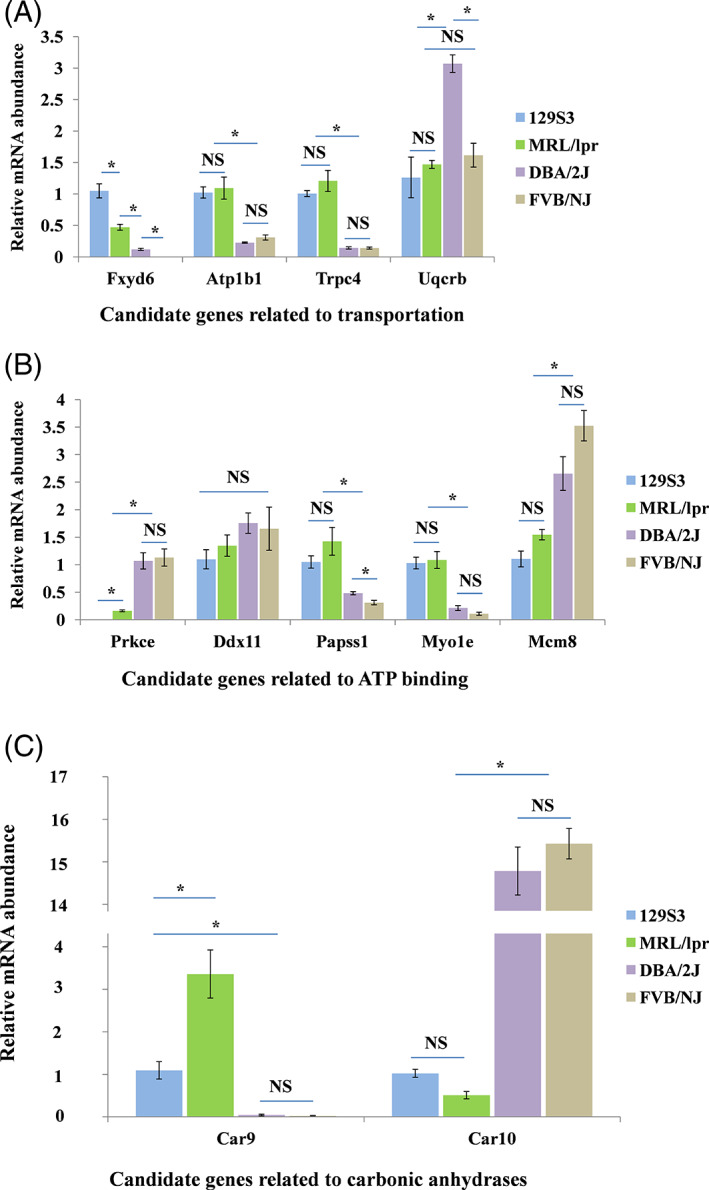
Relative quantification of candidate genes by RT‐qPCR in MII stage oocytes from 129S3, MRL/lpr, DBA/2J, and FVB/NJ inbred mouse strains. (A) Candidate genes related to transportation. (B) Candidate genes related to ATP binding. (C) Candidate genes related to carbonic anhydrases. (*p < 0.05. NS: non‐significant. Data were presented as means ± SEM). RT‐qPCR, real‐time quantitative PCR

The results indicated that 82% (9/11) of the candidate genes show significant difference in abundance between HOC and LOC groups. Specifically, the expression levels of *Atp1b1*, *Car9*, *Fxyd6*, *Myo1e*, *Papss1*, and *Trpc4* were significantly lower. In comparison, the expression levels of *Car10*, *Prkce*, and *Mcm8* were significantly higher in HOC strains based on both microarray and RT‐PCR results. The remaining 18% (2/11) of the candidate genes (*Uqcrb* and *Ddx11*) failed to show any significant difference between HOC and LOC groups by RT‐qPCR result (Figure 2A–C), possibly due to the different sensitivity between the two methods or individual variations from independent samples collected for the two experiments.

### 3.4 Functional study of *Prkce* gene knockout effect on the first cleavage after fertilization

*Prkce*, a gene encoding a novel calcium‐independent protein kinase C isotype—PKCE, attracted our attention among these verified candidate genes. *Prkce* expression was reported to increase significantly in ovaries after sexual maturity, indicating that the gene may be involved in reproductive function.^11^ Moreover, *Prkce* is involved in cell cycle functions in various eukaryotic cells, including cytokinesis,^12^, ^13^ uncoupling chromosome linkage, and activating G1 phase cyclin.^14^, ^15^ However, the effect of *Prkce* on fertilization and the first cleavage has not previously been reported.

**FIGURE 3:**
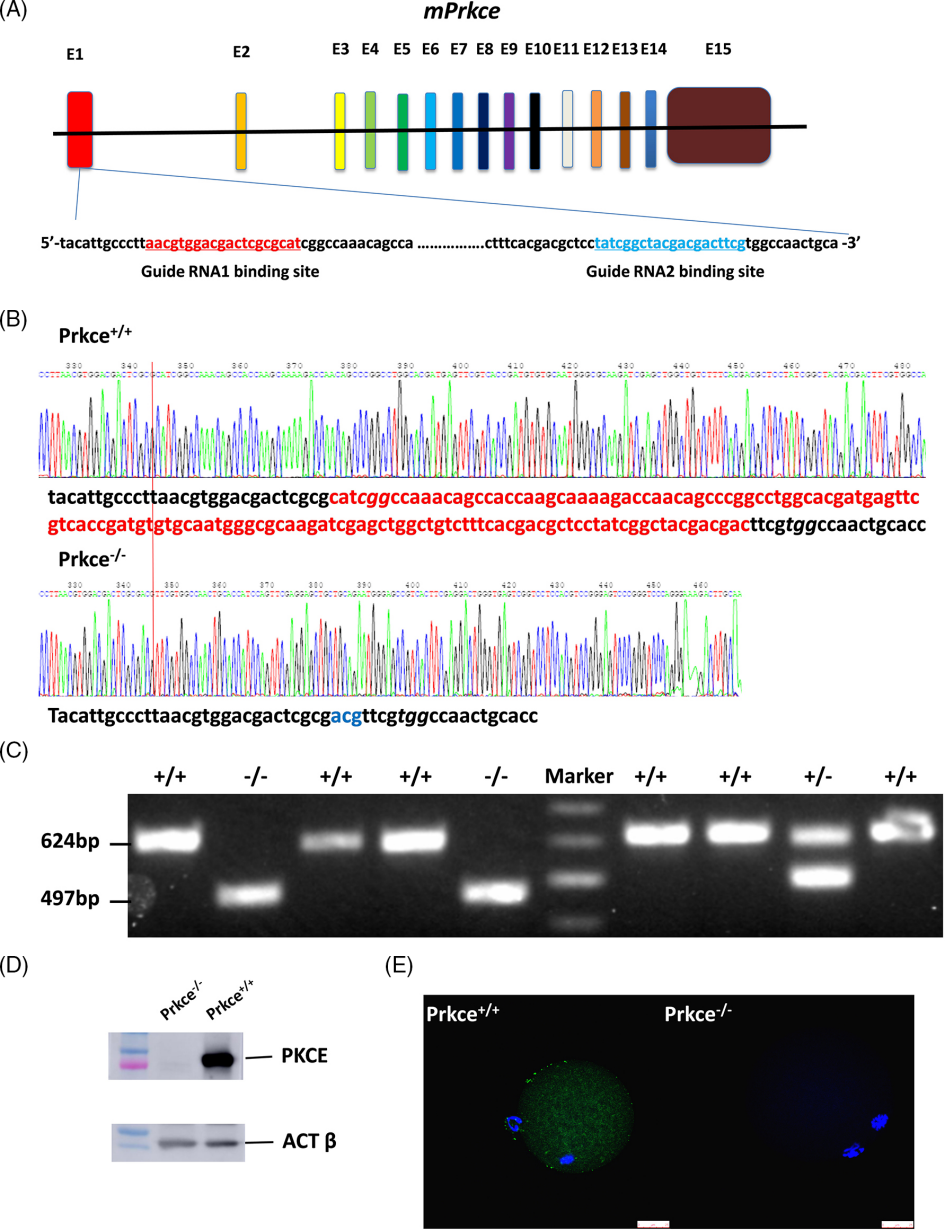
Establishment and validation of *Prkce* knockout mice. (A) Deletion of DNA fragment of exon 1 of *Prkce* via CRISPR/Cas9 method. Guide RNA binding site and schematic diagram of intron and exon structure for *Prkce* are *shown*. Guide RNA 1/2 specifically binding to the exon 1 of *Prkce* are shown with an underline. (B) The sequence of *Prkce* ^*+/+*^ and *Prkce* ^*−/−*^ mice. The red bar shows the sites that differ between *Prkce* ^*+/+*^ and *Prkce* ^*−/−*^ mice. Red characters are the 130 deleted sequences in the *Prkce* ^*−/−*^ knockout mouse, and blue characters are the three inserted sequences in *Prkce* ^*−/−*^ mice. (C) Genotyping of pups by PAGE‐PCR assays. Genomic DNA was extracted from tail samples and subjected to PCR amplification as a template. Every lane represents different mice. *Prkce* wild type (*Prkce* ^*+/+*^): 624 bp; *Prkce* mutant (*Prkce* ^*−/−*^, with 130 bp deleted and 3 bp inserted): 497 bp. (D) Detection of the PKCE protein in ovaries of *Prkce* ^*−/−*^ and *Prkce* ^*+/+*^ mice. (E) Immunofluorescence staining in oocytes of *Prkce* ^*−/−*^ and *Prkce* ^*+/+*^ mice. Green indicates PKCE protein and blue indicates nucleus. PKCE protein is evenly distributed in cytoplasm of MII oocytes. Scale bars are 25 μm. PAGE, polyacrylamide gel elcetrophoresis

To assess the impact of *Prkce* on fertilization and the first cleavage, *Prkce*‐deficient mice were produced via the CRISPR‐Cas9 method. Guide RNA 1/2, specifically targeting the exon 1 of *Prkce* and Cas9 mRNA, was injected into the zygotes (Figure 3A). The *Prkce* knockout mice were identified by PAGE‐PCR assays and DNA sequencing (Figure 3B,C). PCR assay results showed that the homozygous mutant mice (*Prkce* ^*−/−*^) had a 497 bp band, while the wild type (*Prkce* ^*+/+*^) had the expected 624 bp band. Offspring with 130 bp deletion and 3 bp insertion in exon 1 of the *Prkce* gene was obtained. PKCE protein was not detected in the ovaries and oocytes from the *Prkce* ^*−/−*^ mice by Western blotting and immunofluorescence experiment (Figure 3D,E).

**FIGURE 4:**
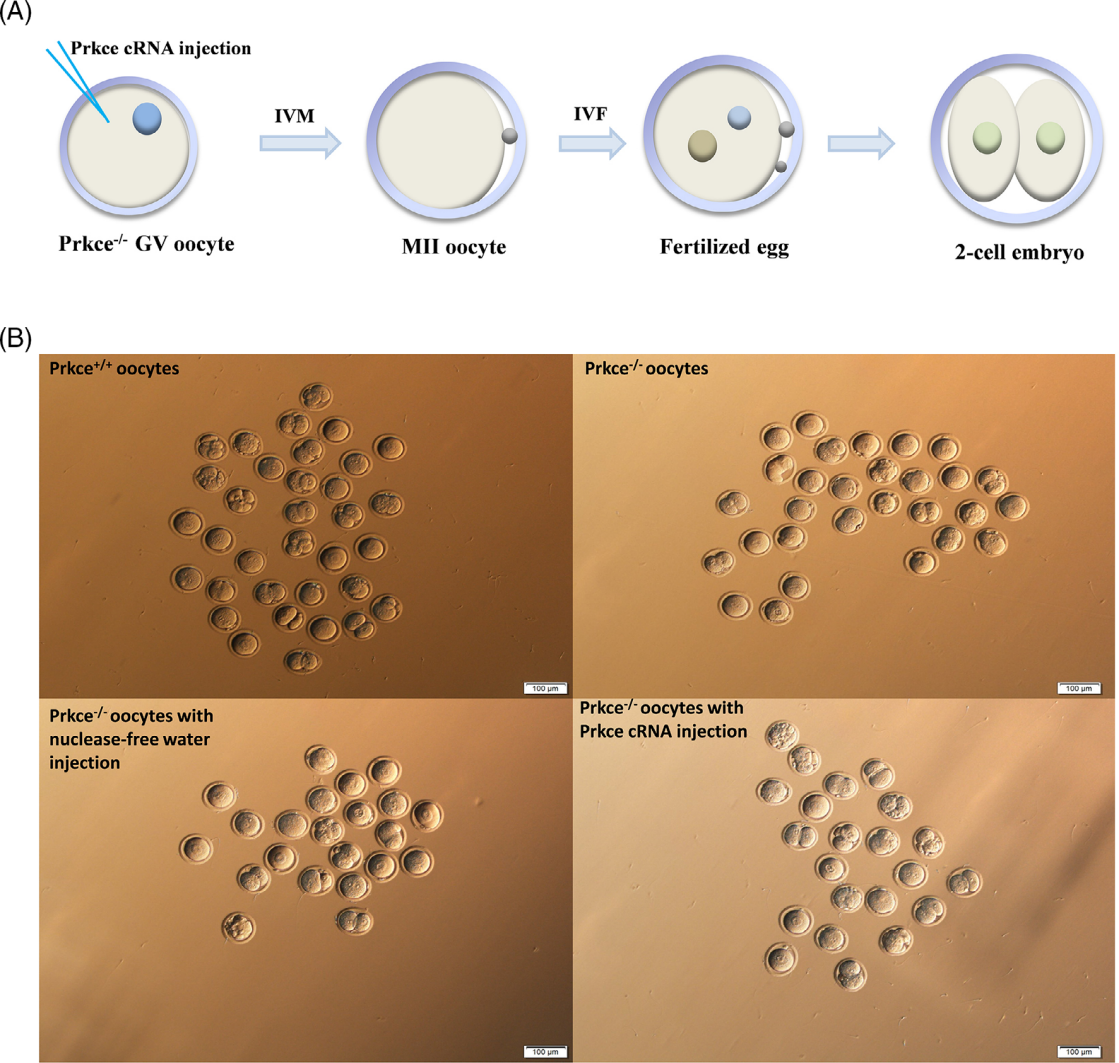
IVF results of *Prkce* knockout oocytes and the effect of *Prkce* cRNA supplementation on *Prkce* knockout oocytes. (A) Scheme for overexpressing the *Prkce* gene. 5′ capped and 3′ polyA‐tailed *Prkce* cRNA were injected into the cytoplasm of *Prkce* ^*−/−*^ GV stage oocytes. After 24 h of incubation in IVM medium, which ensured *Prkce* cRNA to be translated into protein, the MII stage oocytes were carried out with IVF. The fertilization rate and the first cleavage rate were observed 24 h post fertilization. (B) The embryo morphology 24 h after IVM–IVF following *Prkce* cRNA injection into GV oocytes. IVF, *in vitro* fertilization; IVM, *in vitro* maturation

Subsequently, *Prkce* ^*−/−*^ and *Prkce* ^*+/+*^ oocytes matured *in vitro* were used for IVF. The *Prkce* ^*−/−*^ oocytes resulted in a significant decrease in 2‐cell rates (32.4% vs. 80.1% for *Prkce* ^*−/−*^ and *Prkce* ^*+/+*^ oocytes, respectively). No significant difference in the fertilization rate was observed (Table 5, Figure 4B). This data suggests that *Prkce* plays an essential role in the first cleavage.

**TABLE 5:** *In vitro* fertilization results of *Prkce* knockout oocytes and the effect of *Prkce* cRNA supplementation on *Prkce* knockout oocytes

Oocyte status	No. GV oocytes cultured	No. mature oocytes (%)	No. fertilized eggs (%)	No. 2‐cell embryos from fertilized eggs (%)
Non‐injection *Prkce* ^*+/+*^ oocytes	123	109 (88.6%)	72 (66.1%)	58 (80.1%)^a^
Non‐injection *Prkce* ^*−/−*^ oocytes	68	60 (88.2%)	37 (61.7%)	12 (32.4%)^b^
*Prkce* ^*−/−*^ oocytes with nuclease‐free water injection	79	66 (83.5%)	42 (63.6%)	13 (31.0%)^b^
*Prkce* ^*−/−*^ oocytes with *Prkce* cRNA injection	83	68 (81.9%)	43 (63.2%)	33 (76.7%)^a^

*Note*: Values in the same column with different superscripts are significantly different.

To further prove that the *Prkce* gene affects the first cleavage rate, we prepared 5′ capped and 3′ polyA‐tailed *Prkce* cRNA by in vitro transcription and performed a rescue experiment by injecting the *Prkce* cRNA into *Prkce* ^*−/−*^ GV stage oocytes and then carried out IVF (Figure 4A). The first cleavage rate of *Prkce* ^*−/−*^ oocytes supplemented with the *Prkce* cRNA increased significantly (76.7%) which approximates the rate observed in the *Prkce* ^*+/+*^ oocytes group (80.1%). Both of these are significantly higher than the two negative control groups, i.e. non‐injection *Prkce* ^*−/−*^ oocyte controls (32.4%) and *Prkce* ^*−/−*^ oocytes injected with nuclease‐free water controls (31.0%) (Table 5, Figure 4B). The results clearly demonstrated that injection of *Prkce* cRNA into *Prkce* ^*−/−*^ oocytes could rescue the decreased the first cleavage rate of *Prkce* ^*−/−*^ oocytes and further demonstrated that *Prkce* has a critical effect on the first cleavage of fertilized oocytes.

### 3.5 *Prkce* knockout experiment on the initial maternal mRNA pool in the mature oocyte

**FIGURE 5:**
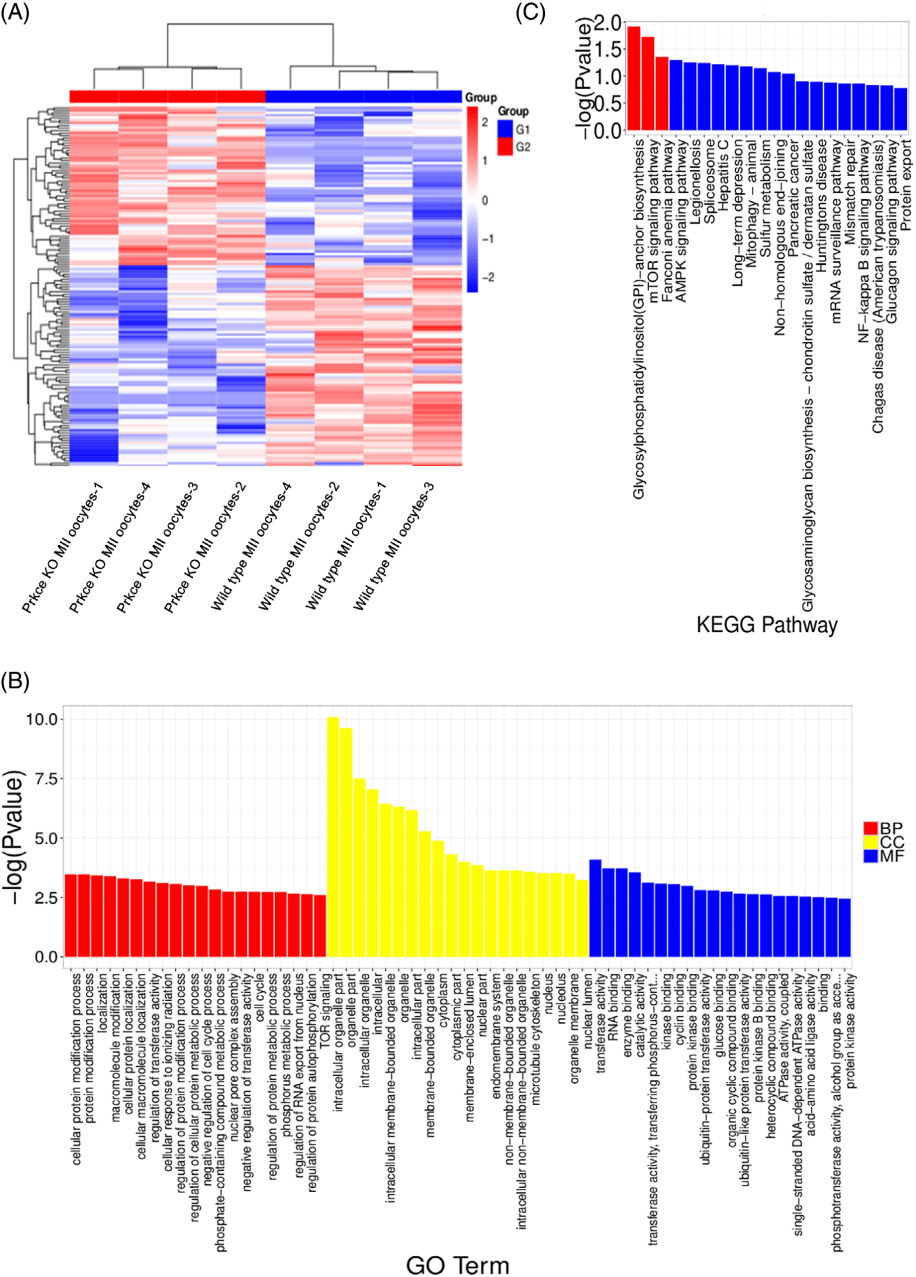
Clustering analysis, Gene Ontology (GO), and KEGG pathway analysis based on the differentially expressed genes (DEGs) between *Prkce* ^*−/−*^ and *Prkce* ^*+/+*^ oocytes. (A) Heat map based on cluster analysis of DEGs. (B) The top 20 GO terms were selected from GO enrichment analysis for DEGs. BP: biological process, CC: cell component, MF: molecular function. (C) The top 20 KEGG pathway enrichment results of DEGs. The red column indicates the pathway with a significant difference (*p*‐value ≤ 0.05), and the blue column indicates the pathway with no significant difference. KEGG, Kyoto Encyclopedia of Genes and Genomes

To investigate how *Prkce* affects the first embryo cleavage, gene expression profiles of *Prkce* ^*−/−*^ MII oocytes and *Prkce* ^*+/+*^ MII oocytes were compared using RNA‐seq data. Based on whole transcriptome analysis, a large set of dysregulated genes in *Prkce* ^*−/−*^ MII oocytes were revealed, including 63 upregulated genes and 80 downregulated genes (Figure 5A, Table S2).

Gene Ontology enrichment analysis uncovered a variety of perturbed biological processes (BP), cellular components, and molecular function. The top 20 GO terms are given in Figure 5B. Some of the top enriched BP are related to the cell cycle, which correlates nicely with the fact that *Prkce* deficiency may interrupt cell‐cycle‐related genes and thus impede the first cleavage.

KEGG pathway analysis was also carried out, and three KEGG pathways were significantly represented (Figure 5C), including the mTOR signalling pathway that was previously reported to be related to oocyte competence.^16^ Therefore, an abnormal abundance of mTOR signalling pathway members induced by the absence of *Prkce* may explain the decreased 2‐cell rate.

## 4 DISCUSSION

This study investigated the differences in expression profiles between MII oocytes from HOC mouse strains and LOC mouse strains by high‐throughput expression analysis. After verification with independent samples by RT‐qPCR, candidate genes that may affect fertilization and the first cleavage of zygote were identified. One of the candidate genes, *Prkce*, previously reported to be involved in mitosis, was selected for further functional investigations.

We utilized inbred mice as their genetic loci were homologous,^17^, ^18^ which could reduce the heterogeneity among different individuals within the same highly inbred strain. The experimental design could be extended in future to a larger number of mouse strains to uncover further knowledge governing the different oocyte competence and some of the mechanisms for 2‐cell block. On a separate note, considering that IVF has been widely used in assisted reproduction, our study focuses on the molecular mechanism affecting 2‐cell rate under IVF conditions. DEGs resulted from our study could be useful candidate genes to study the mechanism and to improve the efficiency of embryo development under *in vitro* condition in future.

Fertilization is a complex process in which sperms recognize and pass through the zona pellucida of mature oocytes, fuse with the oocyte membrane, and activate oocytes.^19^ Mature oocytes contain many maternal genes regulating fertilization, in which *Piga* and *Cd9* are known to be involved in sperm–oocyte fusion.^20^, ^21^ Changes in ion channels and transporters during oocyte maturation give mature oocytes the ability to support fertilization.^22^ Our results demonstrated that some of the DEGs found to differ between LOC and HOC groups are involved in ion transport, such as *Trpc4*, *Atp1b1*, and *Fxyd6*. This suggests that these DEGs may be responsible for low fertility. TRPC4 is a transient receptor potential channel on the cell membrane, and as a non‐selective calcium channel, plays an important role in calcium influx.^23^ Calcium influx is essential for maintaining calcium oscillations, which are necessary for activating fertilized eggs. There has been some evidence indicating that several members of the TRP channel family mediate calcium influx during MZT.^22^ Taken together, *Trpc4* is likely to play an important role in fertilization.

After fertilization, the fertilized eggs undergo a series of cleavage. The first cleavage is most unique because there are minimum transcription and translation activities during the first cleavage,^24^ unlike the mitosis of somatic cells. Therefore, the maternal message pool in the mature oocytes plays an important role in regulating the first cleavage, exampled by *Plk1* which is involved in the regulation of G2/M conversion and oocyte‐specific gene, *Zar1*.^25^, ^26^, ^27^ One candidate gene, *Prkce*, was previously reported to play an essential role in mitosis. PKCE can bind to 14‐3‐3 protein to inactivate RhoA, therefore, providing the power for cell contraction in telophase, which is essential to complete cytokinesis.^12^, ^13^ There is no obvious cell furrow around the equator plate observed in 1‐cell arrested embryos obtained from *Prkce* ^*−/−*^ oocytes, indicating that *Prkce* deletion seems unlikely to affect the cytokinesis at telophase, but more likely to affect the earlier mitotic stages. In *Prkce* ^*−/−*^ mature oocytes, some genes showed abnormal abundance involved in cell cycle regulation, such as *Spag5*, *Fbxo7*, *Tex14*, and others. SPAG5 is an important component of the spindle, which is necessary for normal chromosome segregation and for the cell to enter the anaphase.^28^, ^29^, ^30^ Therefore, *Prkce* is likely to participate in regulating the first cleavage of fertilized eggs by regulating spindle‐related genes.

In conclusion, our study investigated DEGs of mature oocytes between HOC and LOC strains of mice during very early embryo development. Among these, *Prkce* plays a critical role in the first cleavage. The absence of *Prkce* may disturb the abundance of genes related to cell cycle regulation in mature oocytes. This study demonstrated that maternal messages such as *Prkce* could be important for the first cleavage and could serve as a potential marker to characterize high‐quality mature oocytes. The study in time may also help us to better understand the maternal transcripts regulating early embryonic development before ZGA.

## CONFLICT OF INTEREST

The authors have declared that no competing interests exist.

## AUTHOR CONTRIBUTIONS

Shaoqing Zhang, Fanyi Zeng, and Yitao Zeng designed the study. Fanyi Zeng contributed to materials and reagents. Shaoqing Zhang, Xiuli Gong, Qingwen Ma, Qin Cai, Yanwen Chen, Guanheng Yang, Xinbing Guo, Yanwen Chen, and Miao Xu performed experiments. Shaoqing Zhang, Yiye Zhou, and Fanyi Zeng analyzed and interpreted the data. Shaoqing Zhang, Yiye Zhou, and Fanyi Zeng drafted the article. All authors have approved the final version of the submitted manuscript.

## Supporting information


**TABLE S1** Primer sequences for real‐time quantitative polymerase chain reaction

**TABLE S2** List of DEGs between oocytes from Prkce KO and wild type mice

## Data Availability

All related data not included in the manuscript will be availably upon request.

## References

[1] Svoboda P , Franke V , Schultz RM . Sculpting the transcriptome during the oocyte‐to‐embryo transition in mouse. Curr Top Dev Biol. 2015;113:305‐349. 26358877 10.1016/bs.ctdb.2015.06.004

[2] Zeng F , Schultz RM . RNA transcript profiling during zygotic gene activation in the preimplantation mouse embryo. Dev Biol. 2005;283(1):40‐57. 15975430 10.1016/j.ydbio.2005.03.038

[3] Li L , Ping Z , Dean J . Maternal control of early mouse development. Development. 2010;137(6):859‐870. 20179092 10.1242/dev.039487 PMC2834456

[4] Li L , Lu X , Dean J . The maternal to zygotic transition in mammals. Mol Aspects Med. 2013;34(5):919‐938. 23352575 10.1016/j.mam.2013.01.003 PMC3669654

[5] Tong ZB , Gold L , Pfeifer KE , et al. Mater, a maternal effect gene required for early embryonic development in mice. Nat Genet. 2000;26(3):267‐268. 11062459 10.1038/81547

[6] Burns KH , Viveiros MM , Ren YS , et al. Roles of NPM2 in chromatin and nucleolar organization in oocytes and embryos. Science. 2003;300(5619):633‐636. 12714744 10.1126/science.1081813

[7] Labrecque R , Sirard MA . The study of mammalian oocyte competence by transcriptome analysis: progress and challenges. Mol Hum Reprod. 2014;2:103‐116. 10.1093/molehr/gat082 24233546

[8] Byers SL , Payson SJ , Taft RA . Performance of ten inbred mouse strains following assisted reproductive technologies (ARTs). Theriogenology. 2006;65(9):1716‐1726. 16271754 10.1016/j.theriogenology.2005.09.016

[9] Frazer KA , Eskin E , Kang HM , et al. A sequence‐based variation map of 8.27 million SNPs in inbred mouse strains. Nature. 2007;448(7157):1050‐1053. 17660834 10.1038/nature06067

[10] Livak KJ , Schmittgen TD . Analysis of relative gene expression data using real‐time quantitative PCR and the 2(‐Delta Delta C[T]). Methods. 2013;25(4):402‐408. 10.1006/meth.2001.1262 11846609

[11] Tepekoy F , Ustunel I , Akkoyunlu G . Protein kinase c isoforms α, δ and ε are differentially expressed in mouse ovaries at different stages of postnatal development. J Ovarian Res. 2014;7(1):117. 25491605 10.1186/s13048-014-0117-z PMC4271327

[12] Saurin AT , Durgan J , Cameron AJ , Faisal A , Marber MS , Parker PJ . The regulated assembly of a PKCepsilon complex controls the completion of cytokinesis. Nat Cell Biol. 2008;10(8):891‐901. 18604201 10.1038/ncb1749

[13] Saurin AT , Brownlow N , Parker PJ . Protein kinase C epsilon in cell division: control of abscission. Cell Cycle. 2009;8(4):549‐555. 19197162 10.4161/cc.8.4.7653

[14] Brownlow N , Pike T , Zicha D , Collinson L , Parker PJ . Mitotic catenation is monitored and resolved by a PKCε‐regulated pathway. Nat Commun. 2014;5(5):5685. 25483024 10.1038/ncomms6685 PMC4272242

[15] Soh JW , Weinstein IB . Roles of specific isoforms of protein kinase C in the transcriptional control of cyclin D1 and related genes. J Biol Chem. 2003;278(36):34709‐34716. 12794082 10.1074/jbc.M302016200

[16] Guo J , Zhang T , Guo Y , et al. Oocyte stage‐specific effects of MTOR determine granulosa cell fate and oocyte quality in mice. Proc Natl Acad Sci U S A. 2018;115(23):E5326‐E5333. 29784807 10.1073/pnas.1800352115 PMC6003357

[17] Altman PL , Katz DD . Inbred and Genetically Defined Strains of Laboratory Animals. Part 1: Mouse and Rat. Federation of American Societies for Experimental Biology; 1979.

[18] Morse HC . The laboratory mouse ‐ a historical perspective. In: Foster HL , Small JD , Fox JG , eds. The Mouse in Biomedical Research. Academic Press; 1981:1–16.

[19] Yeste M . Boar spermatozoa within the oviductal environment (III): fertilisation. In: Bonet S , Casas I , Holt WV , Yeste M , eds. Boar Reproduction: Fundamentals and New Biotechnological Trends. Springer; 2013:343‐406.

[20] Alfieri JA , Martin AD , Takeda J , Kondoh G , Myles DG , Primakoff P . Infertility in female mice with an oocyte‐specific knockout of GPI‐anchored proteins. J Cell Sci. 2003;116(11):2149‐2155. 12692150 10.1242/jcs.00430

[21] Miyado K , Yamada G , Yamada S , et al. Requirement of CD9 on the egg plasma membrane for fertilization. Science. 2000;287(5451):321‐324. 10634791 10.1126/science.287.5451.321

[22] Ingrid C , Matthias P , Maier TJ , et al. Ion channel function during oocyte maturation and fertilization. Front Cell Dev Biol. 2018;6:63. 29998105 10.3389/fcell.2018.00063 PMC6028574

[23] Nilius B , Flockerzi V . Mammalian transient receptor potential (TRP) cation channels. Preface. Handb Exp Pharmacol. 2014;223(5):v‐vi. 25296415

[24] Abe K , Funaya S , Tsukioka D , et al. Minor zygotic gene activation is essential for mouse preimplantation development. Proc Natl Acad Sci U S A. 2018;115(29):E6780‐E6788. 29967139 10.1073/pnas.1804309115 PMC6055165

[25] Zhao Y , Ai J , Zhang H , Zhu G . Polo‐like kinase‐1 regulates first cleavage of one‐cell embryos in culture during assisted reproduction. Saudi Med J. 2010;31(3):247‐252. 20231927

[26] Baran V , Solc P , Kovarikova V , Rehak P , Sutovsky P . Polo‐like kinase 1 is essential for the first mitotic division in the mouse embryo. Mol Reprod Dev. 2013;80(7):522‐534. 23649868 10.1002/mrd.22188

[27] Tian Y , Yang J , Peng Y , et al. Variation screening of zygote arrest 1 (ZAR1) in women with recurrent zygote arrest during IVF/ICSI programs. Reprod Sci. 2020;27(12):2265‐2270. 32700283 10.1007/s43032-020-00246-y

[28] Mack GJ , Compton DA . Analysis of mitotic microtubule‐associated proteins using mass spectrometry identifies astrin, a spindle‐associated protein. Proc Natl Acad Sci. 2002;98(25):14434‐14439. 10.1073/pnas.261371298 PMC64699 11724960

[29] Gruber J . The mitotic‐spindle‐associated protein astrin is essential for progression through mitosis. J Cell Sci. 2002;115(21):4053‐4059. 12356910 10.1242/jcs.00088

[30] Chu XG , Chen XY , Wan QW , Zheng Z , du Q . Nuclear mitotic apparatus (NuMA) interacts with and regulates Astrin at the mitotic spindle. J Biol Chem. 2016;291(38):20055‐20067. 27462074 10.1074/jbc.M116.724831 PMC5025691

